# Sesn2 gene ablation enhances susceptibility to gentamicin-induced hair cell death via modulation of AMPK/mTOR signaling

**DOI:** 10.1038/cddiscovery.2017.24

**Published:** 2017-05-29

**Authors:** Eliane Ebnoether, Alessia Ramseier, Maurizio Cortada, Daniel Bodmer, Soledad Levano-Huaman

**Affiliations:** 1 Department of Biomedicine, Head and Neck Surgery, University of Basel Hospital, Basel, Switzerland; 2 Department of Otolaryngology, Head and Neck Surgery, University of Basel Hospital, Basel, Switzerland

## Abstract

The process of gentamicin-induced hair cell damage includes the activation of oxidative stress processes. Sestrins, as stress-responsive proteins, protect cells against oxidative stress. Sestrins, particularly Sestrin-2, suppress excessive reactive oxygen species (ROS) accumulation and inhibit mammalian target of rapamycin complex 1 (mTORC1). Thus, we addressed the role of Sestrin-2 in the regulation of sensory hair cell survival after gentamicin exposure. Here, we show that Sestrins were expressed in the inner ear tissues, and Sestrin-2 immunolocalized in sensory hair cells and spiral ganglion (SG). The expression of Sestrin-2 was unchanged, and later downregulated, in gentamicin-treated explants from wild-type mice *in vitro*. Compared with wild-type mice, Sestrin-2 knockout mice exhibited significantly greater hair cell loss in gentamicin-treated cochlear explants. Significant downregulation of phosphorylation of AMP-activated protein kinase alpha (AMPKα) and upregulation of the 70-kDa ribosomal protein S6 kinase (p70S6K) were measured in wild-type cochlear explants exposed to gentamicin compared with their untreated controls. Such regulatory effect was not observed between explants from untreated and gentamicin-treated knockout mice. The gentamicin effect on mTOR signaling was rapamycin-sensitive. Thus, our data provide evidence that Sestrin-2 plays an important role in the protection of hair cells against gentamicin, and the mTOR signaling pathway appears to be modulated by gentamicin during hair cell death.

## Introduction

The organ of Corti (OC) can be damaged by aging, noise exposure, aminoglycoside antibiotics or chemotherapeutic agents. One of the common effects of gentamicin-induced ototoxicity is the activation of oxidative stress.^1^ Administration of antioxidants protects hair cells against ototoxicity.^2^ However, the molecular mechanisms underlying sensory hair cell death and protection are not fully understood.

Sestrins belong to a family of evolutionarily conserved proteins and play a critical role in metabolic homeostasis and in protection against oxidative and genotoxic stress and aging. Three mammalian Sestrin isoforms (Sestrin-1 (Sesn1), Sestrin-2 (Sesn2) and Sestrin-3 (Sesn3)) are known, encoded by three independent genomic loci. Among the three Sestrin isoforms, Sesn2 has been studied most and has an antioxidant function, which influences cell survival.^3,4^ Sesn2 mainly exerts its antioxidant function, attenuating reactive oxygen species (ROS) accumulation, through two pathways: activation of the nuclear factor erythroid 2-related factor (Nrf2) pathway leading to the expression of antioxidant proteins or inhibition of mammalian target of rapamycin complex 1 (mTORC1) activity leading to reduced ER stress or allowing the induction of autophagy.^5^ mTORC1 is sensitive to the immunosuppressive drug rapamycin and is one of two structurally and functionally distinct complexes of mammalian target of rapamycin (mTOR). Sesn1 and Sesn2 have been shown to inhibit mTOR signaling via activation of AMP-activated protein kinase (AMPK), independent of Sestrin redox activity.^6^ Furthermore, ectopic expression of Sesn1 and Sesn2 inhibits mTORC1-dependent phosphorylation of p70-kDa ribosomal protein S6 kinase (p70S6K), the S6 ribosomal protein (S6RP) and 4E-binding protein (4EBP).^6^ The activation of AMPK and inhibition of mTORC1 are disrupted in Sesn2-deficient hepatic cells and liver tissue upon the induction of endoplasmic reticulum (ER) stress.^7^ Moreover, an impaired activation of AMPK has been demonstrated in ischemic hearts from Sesn2 knockout (KO) mice.^8^ Structural information on human Sesn2 was reported recently, helping us to understand the physiological role of Sestrins. The three functionally distinct domains simultaneously inhibit ROS accumulation, regulate mTORC1 activation and bind the amino acid leucine.^9,10^

Sesn2 has been assigned a protective role in preventing age-related pathologies,^11^ maintaining metabolic homeostasis in mouse liver and adipose tissue,^12^ modulating pain processing after peripheral nerve injury^13^ and in the mammalian heart during ischemic insults.^8^ Furthermore, inactivation of Sesn2 improves lung function in a mouse model of chronic obstructive pulmonary disease, which is associated with upregulation of the mTORC1 pathway.^14^

The role of Sesn2 in the inner ear has not yet been investigated. Different stress-inducing agents or insults stimulate the expression of Sesn2 mRNA and/or protein,^7,13^ which then subsequently protects cells against elevated ROS levels.^9^ Therefore, based on the important role of Sesn2 in protecting cells from oxidative stress events and the side effects of gentamicin observed in the inner ear, we hypothesized that Sesn2 might be involved in the regulation of sensory hair cell survival. Here, we demonstrate that Sesn2 via modulation of the AMPK/mTOR pathway is involved in protecting sensory hair cells against gentamicin.

## Results

### Sestrins are expressed in the inner ear of wild-type mice

**Figure 1:**
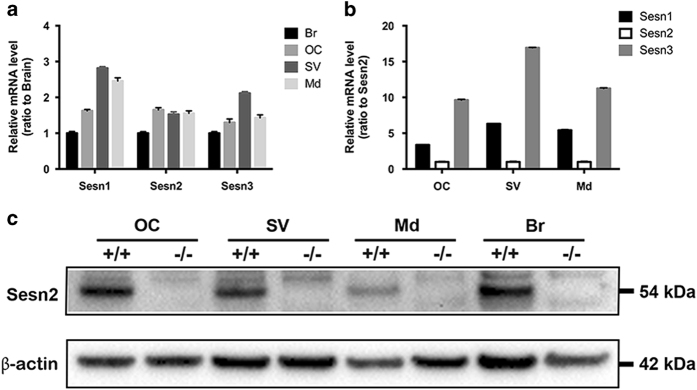
Sestrin expression in the organ of Corti (OC), stria vascularis (SV), modiolus (Md) and brain (Br) of wild-type (WT) mice. (**a **and **b**) mRNA expression was quantified by real-time PCR using the comparative ΔΔCT method. The values were normalized to GAPDH. Sestrin-2 (Sesn2) transcript levels are similar in all inner compartments of the inner ear. The relative mRNA levels are represented as mean+S.D. relative to brain (**a**) or Sesn2 (**b**). RT-PCR reactions were performed in triplicate. Six to seven explants were pooled for each genotype. (**c**) Sesn2 protein expression in inner ear tissues from WT (+/+) and Sesn2 knockout mice (−/−). WT OC had slightly higher Sesn2 protein levels than the other inner ear compartments. Six to seven explants were pooled for each genotype.

First, we investigated the expression levels of all three Sestrins in the mouse cochlea. All three Sestrin transcripts (Sesn1, Sesn2 and Sesn3) were detected in the inner ear compartments of wild-type (WT) mice. After normalization to *β*-actin and taking the brain sample as a calibrator, the transcript level of Sesn1 was 2.8-fold and Sesn3 2.1-fold higher in the stria vascularis than the modiolus and OC, whereas the transcript level of Sesn2 was similar in all inner ear compartments (Figure 1a). When we compared the expression levels of Sesn1 and Sesn3 to Sesn2, Sesn3 mRNA levels were higher than Sesn1 and Sesn2 levels in each of the three compartments (Figure 1b). However, analysis of Sens2 protein in the inner ear compartments found higher protein expression in the OC than the stria vascularis and modiolus, suggesting that Sesn2 protein may play an important role in the inner ear, particularly the OC (Figure 1c).

### Sesn2 is expressed in sensory hair cells and spiral ganglion cells of wild-type mice

**Figure 2:**
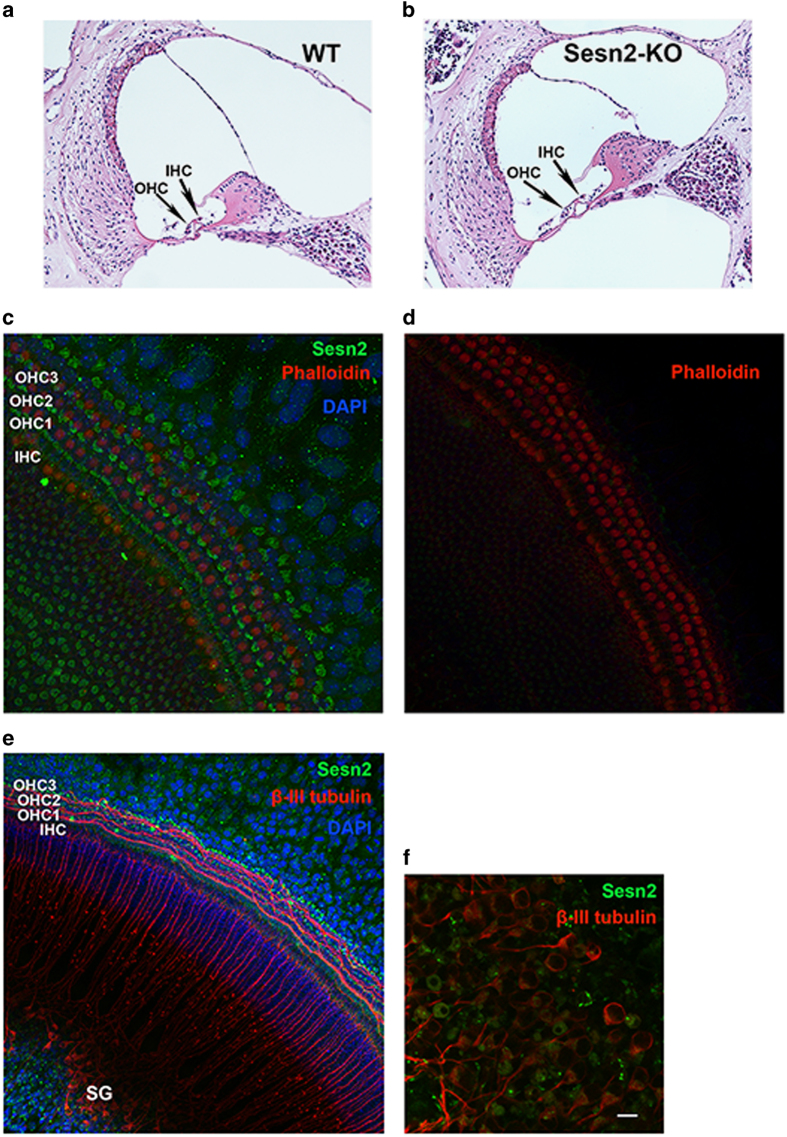
Expression of Sestrin-2 (Sesn2) in mouse sensory hair cells and spiral ganglion. (**a **and **b**) Histology of cochlear sections from WT (**a**) and Sesn2 knockout (KO) mice (**b**) at 5 weeks of age. No visible structural changes were present. (**c** and **d**) Representative confocal image of phalloidin (red) and Sesn2 (green) in the cochlea from P4 mice. Omission of Sesn2 antibody served as a negative control (**d**). (**e **and **f**) Representative confocal images of *β*-III tubulin (red) and Sesn2 (green) in organ explants from WT mice (**e**). A magnified view of spiral ganglion cells (SG) is shown (**f**). Sesn2 immunoreactivity was found in inner hair cells (IHC) and outer hair cells (OHC), as well as the spiral ganglion in the cochlea from P4 mice. Scale bar: (**f**)=10 *μ*m.

To detect possible morphological alterations in the inner ear of Sesn2-KO mice, we performed histochemical staining. Based on hematoxylin and eosin staining of cochlear sections, Sesn2-KO mice exhibited no visible structural alterations in the inner ear compared with WT mice (Figures 2a and b). Using whole-organ explants, Sesn2 immunoreactivity was observed in the hair cells (Figures 2c and e) and spiral ganglion (SG) cells (Figure 2f) of neonatal WT mice, suggesting that Sesn2 participates in physiological processes in the sensory hair cells.

### Gentamicin downregulated Sesn2 protein expression in the late stage of damage

**Figure 3:**
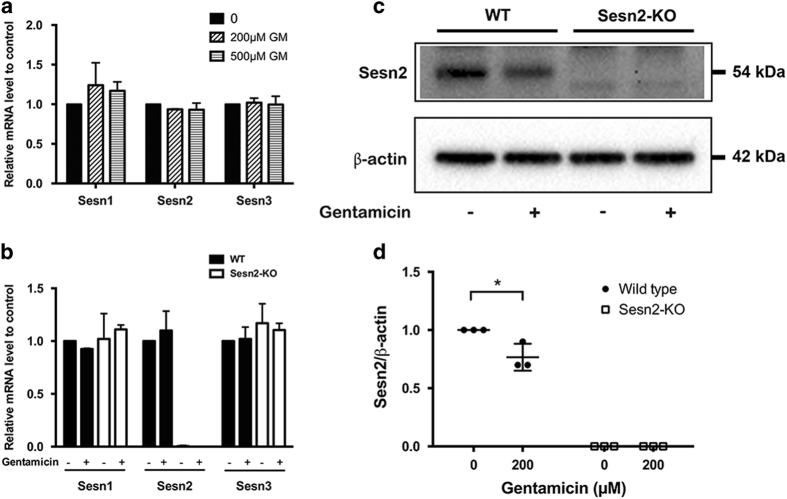
Expression of Sestrin-2 (Sesn2) was diminished after gentamicin exposure. (**a**) Sestrin gene expression in explants from wild-type (WT) mice. Explants were exposed to 200 and 500 *μ*M gentamicin (GM) for 2 h. The values were normalized to GAPDH. (**b**) Sestrin levels in explants from WT and Sesn2 knockout (KO) mice. mRNA expression was quantified by real-time PCR using the comparative ΔΔCT method. Explants were exposed to 200 *μ*M gentamicin for 4 h. The values were normalized to GAPDH. (**c**) Explants were treated with 200 *μ*M gentamicin for 24 h and subjected to western blotting. (**d**) Densitometric analysis of each protein normalized to WT control (untreated). *β*-Actin was used as a loading control. The data are representative of three independent experiments, each with a pool of six explants for each genotype and condition. Values are presented as mean±S.D. *P<0.05.

To determine whether gentamicin exposure alters expression of Sestrins, we measured the transcript levels of Sestrins in organ explants exposed to gentamicin. Transcript levels of Sesn1, Sesn2 and Sesn3 were not affected by different gentamicin concentrations 2 h after exposure in WT mice (Figure 3a). Furthermore, gentamicin exposure did not affect the transcript levels of Sestrins in WT or Sesn2-KO explants after exposure to gentamicin for 6 h *in vitro* (Figure 3b). Sesn1 and Sesn3 transcript levels were not obviously altered upon knockdown of Sesn2. Further analysis of Sesn2 protein expression revealed a significant decrease after 24 h of gentamicin exposure (Figures 3c and d). These results indicate that Sesn2 is maintained during gentamicin-induced stress but downregulated in the late stage of hair cell damage.

### Gentamicin-induced hair cell death was enhanced by knocking out Sesn2

**Figure 4:**
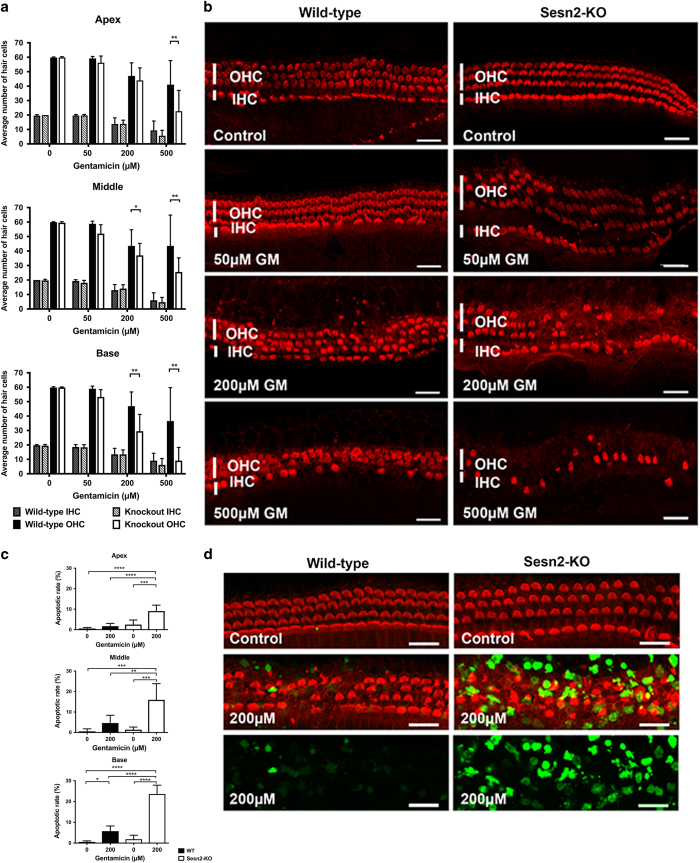
Gentamicin-induced hair cell damage was increased in Sestrin-2 (Sesn2) knockout (KO) mice. (**a**) Quantification of inner hair cell (IHC) and outer hair cell (OHC) survival in wild-type (WT) and Sesn2-KO mice. Explants were treated with different concentrations of gentamicin for 24 h. At 200 *μ*M gentamicin, hair cell damage differed significantly between WT and Sesn2-KO mice at the basal and middle cochlear turns. At least six explants of each genotype and condition were used. (**b**) Representative confocal images of phalloidin-stained hair cells from the basal cochlear turn treated with 200 *μ*M gentamicin for 24 h. Gentamicin induced more hair cell damage in Sesn2-KO mice than in WT mice. Scale bar=50 *μ*m. (**c** and **d**) Quantification of apoptotic rate (**c**) in WT (black) and Sesn2-KO (white) mice and representative confocal images of TUNEL and phalloidin double-stained hair cells (**d**; basal turn). Explants were treated with 200 *μ*M gentamicin for 24 h. At least six explants of each genotype and condition were used. Scale bar=20 *μ*m. Values are presented as mean+S.D. ****P<0.0001, ***P<0.001, **P<0.01, *P<0.05.

To assess the potential role of Sesn2 in gentamicin-induced hair cell death, WT and Sesn2-KO explants were exposed to different gentamicin concentrations for 24 h *in vitro* (Figures 4a and b). Hair cell survival rates in all regions were similar in untreated explants from WT and Sesn2-KO mice. With increasing concentrations of gentamicin, inner hair cell loss increased, but no differences were observed between WT and Sesn2-KO mice along the whole organ. Outer hair cell loss also increased with increasing concentrations of gentamicin, but Sesn2-KO demonstrated greater hair cell loss at 200 and 500 *μ*M gentamicin. At 200 *μ*M gentamicin, mean hair cell survival in the basal region of WT mice was 46.88±9.85, whereas hair cell survival was significantly decreased in Sesn2-KO mice (29.5±11.71). Similar results were obtained from the middle region, as hair cell survival was 43.54±11.12 in WT mice *versus* 37.0±8.29 in Sesn2-KO mice. No differences were observed between WT and Sesn2-KO mice regarding hair cell loss in the apical regions. At the highest gentamicin concentration used in this study, hair cell loss was visible in all regions, and greater hair cell loss was observed in explants from Sesn2-KO mice compared with explants from WT mice. Because most of the hair cells were lost at this high gentamicin concentration, we decided to use 200 *μ*M gentamicin to study the expression of genes involved in the mechanism underlying hair cell damage. The base and middle regions of the explants were used for further experiments.

To confirm that hair cell death occurs through apoptosis, TUNEL assay was performed on WT and Sesn2-KO explants cultured in the presence or absence of gentamicin for 24 h *in vitro*. More TUNEL-positive hair cells were observed in explants from Sesn2-KO mice than from WT mice after gentamicin exposure (Figures 4c and d), suggesting that most of the cells died via apoptosis. These results demonstrate that Sesn2 ablation sensitized hair cells to gentamicin.

### Gentamicin modulated p70S6K and pAMPK

**Figure 5:**
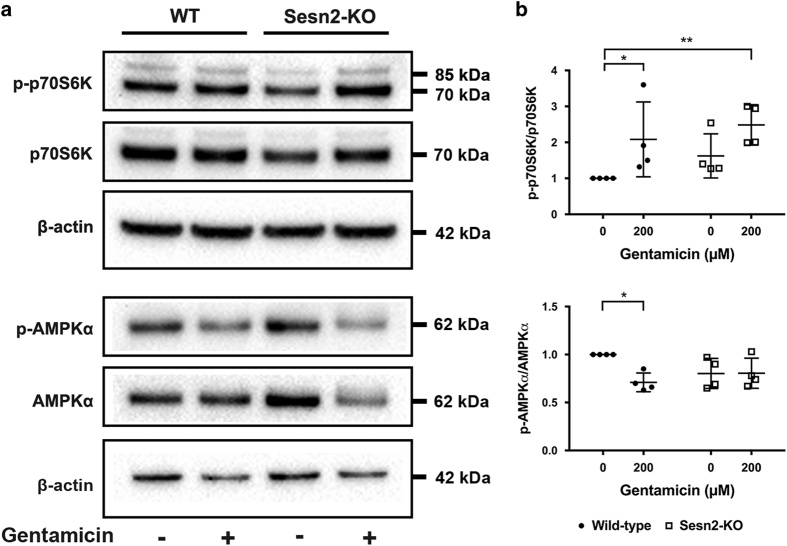
Treatment with gentamicin-induced inactivation of AMPKα and activation of p70S6K. (**a**) Reciprocal modulation of AMPKα and p70S6K was observed in wild-type mice (WT), but not in Sestrin-2 (Sesn2) knockout (KO) mice. Explants were treated with 200 *μ*M gentamicin for 24 h and subjected to western blotting. (**b**) Densitometric analysis of each protein normalized against WT control (untreated). *β*-Actin was used as a loading control. The data are representative of four independent experiments, each with a pool of six explants for each genotype and condition. Values are presented as mean±S.D. **P<0.01, *P<0.05.

To investigate the molecular mechanism underlying hair cell sensitivity to gentamicin, we analyzed the expression of AMPKα and p70S6K in cultured OC explants after gentamicin exposure for 24 h *in vitro* (Figures 5a and b). The phosphorylation status of p70S6K is commonly used to assess mTORC1 activity. At this late stage of hair cell damage after gentamicin exposure, we observed an inactivation of AMPKα and activation of p70S6K in WT mice. However, the effect of gentamicin was not observed in Sesn2-KO mice; activation of p70S6K was greater in untreated and treated Sesn2-KO explants than in untreated WT explants, with a significant difference between treated Sesn2-KO and WT control (*P*<0.01). These results indicate that the mTOR signaling pathway is involved in the process of gentamicin-induced hair cell damage and Sesn2 ablation does not prevent mTORC1 activation.

### Rapamycin attenuated gentamicin-induced hair cell damage

**Figure 6:**
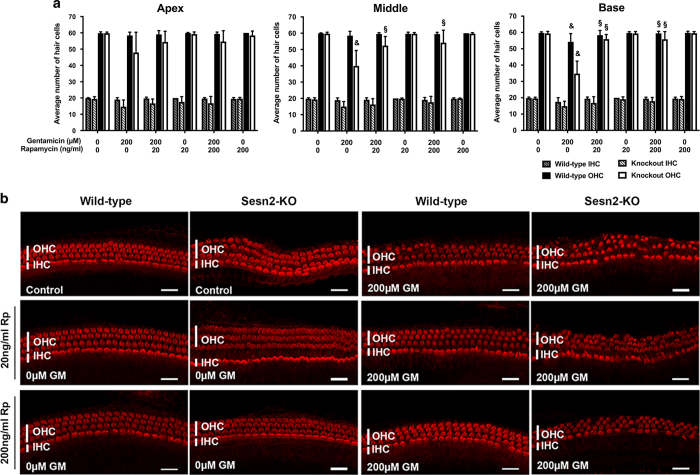
Rapamycin protected hair cells against gentamicin. (**a**) Quantification of inner hair cell (IHC) and outer hair cell (OHC) survival in wild-type and Sesn2-KO mice. Explants were treated with 200 *μ*M gentamicin in the absence or presence of 20 and 200 ng/ml rapamycin for 24 h. The addition of rapamycin to gentamicin increased the number of surviving hair cells in explants from the basal and middle cochlear turns of Sesn2-KO mice. At least five explants of each genotype and condition were used. Values are presented as mean+S.D. ^§^Significant compared with gentamicin-treated sample of the same genotype; ^&^significant compared with control sample of the same genotype. (**b**) Representative confocal images of phalloidin-stained hair cells from the basal cochlear turn treated with 200 *μ*M gentamicin in the presence or absence of rapamycin (Rp) for 24 h. Scale bar=50 *μ*m.

If Sesn2 exerts its otoprotective effect by modulating mTORC1, inhibition of mTORC1 should influence hair cell survival in Sesn2-KO mice after gentamicin exposure. Explants were pre-treated with the mTOR inhibitor rapamycin, followed by gentamicin treatment with and without rapamycin for 24 h. As expected, the addition of rapamycin to gentamicin-treated Sesn2-KO explants significantly increased hair cell survival in basal regions (55.9±2.85 for 20 ng/ml rapamycin and 55.8±4.67 for 200 ng/ml rapamycin) compared with those treated with gentamicin alone (34.75±7.67) (Figures 6a and b). Similar results were obtained from the middle regions of Sesn2-KO explants; hair cell survival was 39.95±9.47 in gentamicin-treated explants *versus* 52.4±5.60 in explants treated with gentamicin plus 20 ng/ml rapamycin or 54.2±7.64 in explants treated with gentamicin plus 200 ng/ml rapamycin.

Despite less hair cell damage by gentamicin in WT explants (54.28±5.0) compared with previous experiments with serial dilutions of gentamicin (46.88±9.85 for 200 *μ*M gentamicin), the addition of rapamycin significantly increased the number of surviving hair cells in the basal region of WT explants compared with those treated with gentamicin alone. These results demonstrate that inhibition of mTOR protects hair cells against gentamicin damage *in vitro*.

## Discussion

Gentamicin-induced ototoxicity consists of multiple intracellular events that implicate survival and apoptotic activities. Oxidative stress in the inner ear is triggered by gentamicin, inducing hair cell damage.^1,2^ All members of the Sestrin family are induced by oxidative stress, and the induction mechanisms differ between their members.^15^ Sesn2 exerts several functions, including a reduction of intracellular ROS levels and regulation of AMPK and mTORC1 signaling.^4,9^ Taking advantage of the availability of Sesn2-KO mice, we investigated the role of Sesn2 in sensory hair cells under stress conditions using gentamicin treatment.

Expression of all three Sestrin genes has been detected in most human and mouse tissues at different levels; Sesn2 is expressed in several tissues, including kidney, liver, lungs and leukocytes.^16^ However, Sestrin gene expression in the inner ear has not been reported. In the present study, we found basal expression of Sestrins in all inner ear compartments. Sesn2 mRNA was expressed at similar levels across the compartments, whereas Sens2 protein expression was higher in OC lysate than in the other compartments. Similar results for all three isoforms have been found in HepG2 cells.^7^ In agreement with our data obtained from real-time PCR and immunoblotting, immunostaining of Sesn2 was observed in the hair cells and SG of cultured explants from WT mice. Sesn2 immunostaining seemed to be stronger in the third row of outer hair cells than in the other rows. Increased expression of Sesn2 in these cells may explain the greater resistance of the cells compared with those of the first row. These data demonstrate, for the first time, the expression of Sestrins, particularly Sesn2, in the inner ear.

The abundance of Sestrins in resting cells is relatively low, but stress stimuli induce Sesn2 expression by activating several transcription factors.^15^ In contrast to findings in other tissues and in the presence of stress stimuli,^7,13^ transcript levels of Sesn2 did not increase during the early drug exposure. Indeed, Sesn2 protein expression diminished after 24 h of gentamicin exposure. One possible explanation for this lack of response could be that the induction of Sesn2 expression by gentamicin in the OC may be an early, transient, and not prolonged effect, if we consider the pharmacokinetic mechanism of gentamicin.^17^ Gentamicin is taken up into sensory hair cells very rapidly and causes excessive ROS production, which leads to a redox imbalance and may trigger cell death.^17,18^ Another possible explanation could be that Sesn2 is constitutively expressed in the OC, as shown in our immunoblotting results, and no further increase is involved in the hair cell damage process.

Our results indicate that the hair cells of Sesn2-KO mice were more sensitive to gentamicin exposure than those of WT mice, and the degree of hair cell loss was dependent on the gentamicin concentration. Our findings are in agreement with previous studies that demonstrated a protective effect of Sestrins against stress insults.^11,19,20^ Sesn2-KO mice have exacerbated neuropathic pain behavior with increased ROS levels.^13^ In addition, ablation of Sesn2 has been shown to inactivate Nrf2 and increase the susceptibility of the liver to oxidative damage.^19^ Furthermore, Sesn2-dependent AMPK activation attenuates fibrotic injury in diabetes.^20^ Other supporting data come from a study using Nrf2-KO mice that suggested that Nrf2 protects hair cells against gentamicin *in vitro* by upregulating antioxidant enzymes.^21^ Sestrins positively regulate the Nrf2 pathway,^5^ and Sesn2 is required for the Nrf2-mediated oxidative stress response pathway.^22^ Therefore, these findings indicate that Sesn2 signaling is involved in protecting hair cells against gentamicin toxicity.

Notably, although Sesn2-KO explants still expressed Sesn1 and Sesn3, more hair cell loss was observed in Sesn2-KO explants than in WT explants. It seems that the loss of Sesn2 is not compensated for by the expression of Sesn1 and Sesn3. Thus, the absence of Sesn2 affects the sensitivity of hair cells to gentamicin.

To identify the mechanism underlying gentamicin-induced hair cell damage, we assessed protein expression in the stress-responsive pathway associated with Sesn2. Information on the protein structure of human Sesn2 has contributed enormously to understanding its distinct functions in the inhibition of ROS accumulation through the modulation of Nrf2 and mTOR activation.^9^ Thus, the specific molecular events in response to insults in particular organs need to be described. Here, we show that gentamicin significantly downregulated pAMPK in WT explants and upregulated p-p70S6K in Sesn2-KO and WT explants. Our results are consistent with previous studies in which Sesn2-KO mice developed chronic ER stress, which was reversed by adding an AMPK activator.^7^ Moreover, larger myocardial infarcts were observed in Sesn2-KO hearts compared with control hearts, and cardiac AMPK was impaired in Sesn2-KO mice.^8^ Sesn2-AMPK activation also protects mitochondrial function against metabolic stress.^23^ In addition, acoustic overstimulation activates AMPK in the cochlear spiral ligament,^24^ protecting the inner ear from auditory trauma.^25^ Sestrins contribute to regulation of the AMPK/mTORC1 signaling pathway, and their activities are critical for maintaining the basal autophagy that removes dysfunctional mitochondria, thereby decreasing pathogenic amounts of ROS.^15^ Therefore, genetic loss of Sesn2 perturbed the regulation of the Sesn2/AMPK/mTORC1 pathway and the cells, in the absence of Sesn2, failed to inhibit p-p70S6K, resulting in increased hair cell loss after gentamicin exposure.

Interestingly, the mTOR inhibitor rapamycin protected sensory hair cells against gentamicin. Evidence of the protective effect of Sesn2 via inhibition of mTORC1 activity has been reported previously. For example, Sesn2 exerts its protective role by inhibiting mTOR via AMPK in glucose-depleted cells.^26^ In addition, this finding is consistent with a recent study by Kim *et al*.,^27^ who demonstrated that activation of autophagy flux by rapamycin protects against GM-induced hearing loss. Furthermore, rapamycin attenuates noise-induced hair cell loss by reducing oxidative stress.^28^ Notably, the rapamycin concentrations in the nanomolar range used in the present study are similar to previous studies that reported protective effects,^7,26^ but contrast our early publication, in which exposure to high concentrations of rapamycin resulted in hair cell damage.^29^ According to our current results, gentamicin induced hair cell damage via mTOR activation.

In summary, our data show that genetic ablation of Sesn2 enhances the sensitivity of hair cells to gentamicin. Furthermore, the process of gentamicin damage primarily involves Sesn2 via regulation of the AMPK/mTOR pathway. Therefore, these findings indicate the importance of the protective role of Sesn2 in gentamicin-induced stress. Even though the precise mechanism underlying the action of Sesn2 is not yet clear, our study takes a first step towards understanding the role of Sestrins in the inner ear and points to the Sestrin/mTOR pathway as a molecular mechanism of ototoxicity that should be investigated further.

## Materials and methods

### Antibodies

Primary antibodies were used at the following dilutions: p-p70S6K at 1:500, p70S6K at 1:1000, pAMPKα at 1:500, AMPKα at 1:1000 (all from Cell Signaling, Bioconcept, Allschwil, Switzerland), Sesn2 at 1:1000 (Proteintech, Lubio Science, Switzerland), class III *β*-tubulin 1:500 (Covance, Geneva, Switzerland) and *β*-actin at 1:5000 (Abcam, Labforce AG, Nunningen, Switzerland). AlexaFluor 568 phalloidin and AlexaFluor-conjugated secondary antibodies were purchased from Thermo Fisher Scientific (Reinach, Switzerland).

### Mice and genotyping

Experiments were performed with 4- to 5-day-old WT and Sesn2-KO mice, RRJ141 (RRJ141/Sesn2^*Gt(RRJ141)Byg*^). The Sesn2-KO mice (kindly provided by Prof. Harald von Melchner, University of Frankfurt Medical School) were maintained on a C57Bl6/J background. PCR amplification of genomic DNA was used to identify mouse genotypes. The animals were housed in pathogen-free conditions at the animal facility of the Department of Biomedicine of the University Hospital Basel. All animal experiments were conducted with the approval of the Animal Care Committee of Canton Basel City, Switzerland.

### Preparation of organs for culture

Mice were decapitated and the cochlea dissected from the skull in cold 1× PBS. The OC explants were placed in Dulbecco’s modified Eagle’s medium supplemented with 10% fetal bovine serum, 25 mM HEPES and 30 U/ml penicillin (all chemicals from Sigma Aldrich Chemie GmbH, Steinheim, Germany, unless otherwise indicated). Explants were incubated at 37 °C and 5% CO_2_ overnight. After recovery, explants were exposed to gentamicin for 2 and 6 h to assess RNA expression or 24 h to assess hair cell damage and protein expression or for TUNEL assay. To evaluate the effects of mTOR inhibition on the cochlear explants, they were pre-treated overnight with rapamycin and then treated with gentamicin in the presence or absence of rapamycin for 24 h.

### RNA analysis

For quantitative real-time PCR, six to seven fresh dissected OC, stria vascularis, modiolus and brain explants were used for each genotype. In the case of treated samples, six OC explants without the apex region were used for each condition and each genotype. Dissected organs were stored in RNA stabilization reagent (Qiagen, Hombrechtikon, Switzerland) at −20 °C until needed. Total RNA was extracted using an RNeasy Plus Mini kit (Qiagen) according to the manufacturer’s instructions. RNA concentration and purity were determined using the Nanodrop ND-1000 (Thermo Fisher Scientific). cDNA synthesis was carried out using a high-capacity cDNA reverse transcription kit (Applied Biosystems, Thermo Fisher Scientific). The TaqMan gene expression assays for mouse Sesn1 (Mm01185732_m1), Sesn2 (Mm00460679_m1), Sesn3 (Mm01171504_m1) and GAPDH (Mm99999915_g1) were purchased from Applied Biosystems. Quantitative PCR was performed using TaqMan gene expression assays and the TaqMan Fast Advanced Master Mix kit (Applied Biosystems) on an ABI 7500 Fast Real-Time PCR instrument (Applied Biosystems). Each reaction was performed in triplicate. Relative mRNA levels were determined using the 2−ΔΔCT method and normalized to the housekeeping gene *GAPDH*.

### Immunofluorescence

The entire OC together with SG was placed on poly-lysine and laminin-coated Ibidi chambers (Ibidi, Martinsried, Germany), preventing explant removal during staining. After overnight incubation, explants were fixed with 4% paraformaldehyde (PFA) in PBS (Santa Cruz Biotechnology, Inc., LabForce AG, Muttenz, Switzerland), permeabilized and blocked with 2.5% Triton X-100 and 10% FBS in PBS. Organs were incubated overnight with primary antibodies and successively incubated with conjugated secondary antibodies. Omission of primary antibody served as a negative control. Images were acquired using a Nikon A1R laser confocal microscope (Nikon AG Instruments, Egg, Switzerland).

### Histology

Paraffin sections of mouse ears were prepared and stained using standard histological procedures. Mice were deeply anesthetized and the inner ears quickly removed from the skull and fixed in 4% PFA at 4 °C overnight. After decalcification in 10% EDTA, samples were embedded in paraffin (TP1020 benchtop tissue processor; Leica Biosystems, Heerbrugg, Switzerland), sectioned and stained with hematoxylin and eosin (Continuous Linear Stainer COT20; Medite GmbH, Burgdorf, Germany).

### Hair cell counting

At the end of each experiment, OC explants were fixed in 4% PFA, permeabilized and stained with AlexaFluor 568 phalloidin. Phalloidin-labeled hair cells were visualized using a Nikon A1R laser confocal microscope with a ×20 lens (Nikon AG Instruments). Surviving inner and outer hair cells were counted in sections corresponding to 20 inner hair cells at three randomly selected regions of the basal, middle and apical turns of each cochlea. Cells were considered missing if there was a gap in the normal ordered array of hair cells. The results are presented as the number of surviving hair cells per cochlear turn. At least five OC explants for each genotype and condition were examined.

### Apoptotic cell staining

At the end of each experiment, OC explants were fixed in 4% PFA, permeabilized and DNA fragments fluorescence *in situ* end-labeled for DeadEnd fluorometric TUNEL assay according to the manufacturer’s instructions (Promega, Madison, WI, USA). Apoptotic hair cells were identified using a Nikon A1R laser confocal microscope with a ×20 lens (Nikon AG Instruments). The apoptosis rate was determined by counting TUNEL-positive cells divided by the total number of cells over a 200-*μ*m distance in the basal, middle and apical turns of each cochlea. The fields were selected randomly.

### Immunoblotting

At least six OC explants were used for each condition and each genotype. After treatment, the explants were washed with 1×PBS and immediately transferred into T-PER lysis buffer (Thermo Fisher Scientific) containing protease and phosphatase inhibitors (Roche Applied Science, Rotkreuz, Switzerland). The proteins were separated by electrophoresis, blotted onto a PVDF membrane, and probed with primary antibodies and the corresponding peroxidase-conjugated secondary antibodies. The bands were visualized by chemiluminescence using West Femto Super Signal (Thermo Fisher Scientific). Immunoblots were always analyzed for the phosphorylated forms of proteins first, then stripped with Restore PLUS Western Blot Stripping Buffer (Thermo Fisher Scientific) and re-probed for the corresponding total protein. *β*-Actin was used as a loading control. The relative densities of specific proteins were calculated using ImageJ software (National Institutes of Health, Bethesda, MD, USA) and normalized against untreated WT control.

### Statistical analysis

Data analysis was performed using GraphPad Prism 6.0 software (GraphPad software, La Jolla, CA, USA). Hair cell damage was analyzed between groups using two-way analysis of variance followed by Tukey’s multiple comparisons test. Immunoblots were analyzed using the non-parametric Kruskal–Wallis test followed by Dunnett’s test. All values are expressed as mean+S.D. *P*-values <0.05 were considered significant.
